# Characteristics of Real-Time, Non-Critical Incident Debriefing Practices in the Emergency Department

**DOI:** 10.5811/westjem.2016.10.31467

**Published:** 2016-12-05

**Authors:** Nur-Ain Nadir, Suzanne Bentley, Dimitrios Papanagnou, Komal Bajaj, Stephan Rinnert, Richard Sinert

**Affiliations:** * OSF St. Francis Medical Center, University of Illinois College of Medicine at Peoria, Department of Emergency Medicine, Peoria, Illinois; † Elmhurst Hospital Center, Icahn School of Medicine at Mount Sinai, Department of Emergency Medicine and Department of Medical Education, Elmhurst, New York; ‡ Thomas Jefferson University Hospital, Department of Emergency Medicine, Philadelphia, Pennsylvania; § Jacobi Medical Center, Department of Obstetrics and Gynecology, New York, New York; ¶ Kings County Hospital and SUNY Downstate Medical Center, Department of Emergency Medicine, New York, New York

## Abstract

**Introduction:**

Benefits of post-simulation debriefings as an educational and feedback tool have been widely accepted for nearly a decade. Real-time, non-critical incident debriefing is similar to post-simulation debriefing; however, data on its practice in academic emergency departments (ED), is limited. Although tools such as TeamSTEPPS® (Team Strategies and Tools to Enhance Performance and Patient Safety) suggest debriefing after complicated medical situations, they do not teach debriefing skills suited to this purpose. Anecdotal evidence suggests that real-time debriefings (or non-critical incident debriefings) do in fact occur in academic EDs;, however, limited research has been performed on this subject. The objective of this study was to characterize real-time, non-critical incident debriefing practices in emergency medicine (EM).

**Methods:**

We conducted this multicenter cross-sectional study of EM attendings and residents at four large, high-volume, academic EM residency programs in New York City. Questionnaire design was based on a Delphi panel and pilot testing with expert panel. We sought a convenience sample from a potential pool of approximately 300 physicians across the four sites with the goal of obtaining >100 responses. The survey was sent electronically to the four residency list-serves with a total of six monthly completion reminder emails. We collected all data electronically and anonymously using SurveyMonkey.com; the data were then entered into and analyzed with Microsoft Excel.

**Results:**

The data elucidate various characteristics of current real-time debriefing trends in EM, including its definition, perceived benefits and barriers, as well as the variety of formats of debriefings currently being conducted.

**Conclusion:**

This survey regarding the practice of real-time, non-critical incident debriefings in four major academic EM programs within New York City sheds light on three major, pertinent points: 1) real-time, non-critical incident debriefing definitely occurs in academic emergency practice; 2) in general, real-time debriefing is perceived to be of some value with respect to education, systems and performance improvement; 3) although it is practiced by clinicians, most report no formal training in actual debriefing techniques. Further study is needed to clarify actual benefits of real-time/non-critical incident debriefing as well as details on potential pitfalls of this practice and recommendations for best practices for use.

## INTRODUCTION

The emergency department (ED) is a complicated teaching environment. Prolonged patient waiting times, frequent interruptions, a diverse set of learners and a variety of emergent, often unpredictable clinical cases compounded with understaffing and limited resources represent the major barriers to effective bedside teaching and provision of feedback to trainees. This challenging learning environment makes a strong argument for ED-specific teaching and learning strategies.^1^–^3^ Anecdotal reports suggest that one teaching tool and feedback strategy being employed by emergency medicine (EM) faculty is real-time, non-critical incident debriefing.

Real-time feedback during a clinical shift in the ED is an important component of a resident physician’s medical education and can have a profound impact on clinical practice.^2^–^5^ Despite this, many residents feel they do not get adequate or useful feedback during their clinical shifts. Specific, tailored, learner-centered feedback is crucial but rarely performed.^2^–^5^

Debriefing is an educational tool based on the principles of adult learning theory that uses a simulated (or real) medical event to generate a discussion of the teachable moments within that event.^6^ Debriefings are critical to healthcare education because that is usually where the critical process of feedback occurs and where learning is often clarified and translated into “take-home points” and guidelines for future practice.^7^,^8^ An example of such an event would be a resident physician encountering a challenging, agitated patient. The teachable opportunity would include a debriefing of the difficulties encountered by the resident and what went smoothly versus what could have been performed differently. Debriefing can be viewed as a conversation about a medical event, where any observed clinical performance gaps are addressed.^9^ Learners are asked open-ended questions in order to clarify their individual thought processes and are also asked to self-critique their performance.^11^,^13^,^14^ By promoting constructive self-critique and self-evaluation, medical debriefing instills practices of life-long learning, considered to be important elements of “practice-based learning,” one of the six core medical education competencies required by the Accreditation Council of Graduate Medical Education..^15^

Research has clearly established the importance of feedback. Debriefing builds on many tenets of feedback including recommendations that it should be timely, specific, tailored, and learner centered.^11^,^13^–^14^ Most of this research, however, has been conducted in simulated environments. With the advent of communication tools such as TeamSTEPPS^16^ (Team Strategies and Tools to Enhance Performance and Patient Safety), debriefing is promoted as a means of self-reflection in order to lead to systems and process improvement.

## METHODS

We recruited four EM residency programs for the purposes of this study. These four programs were chosen because they are large, high-volume, academic teaching hospitals within the city of New York. We contacted residency leadership from each hospital and obtained permission to distribute a questionnaire to EM staff. Questionnaire design commenced with a PubMed literature search using the terms “medical debriefing,” “simulation debriefing,” “non-critical incident debriefing” and “real-time debriefing.” We then identified major landmark articles on medical educational debriefing practices, techniques, and skills. “Critical incident debriefing” and similar psychological debriefing articles were excluded. Based on the literature search, we drafted a questionnaire examining basic characteristics of debriefing.

We identified EM educators and simulation debriefing experts based on their respective research publications and/or involvement in the fields of EM and healthcare simulation and invited them to participate in a Delphi panel for further refinement of the questionnaire. Feedback from the Delphi panel of six experts was incorporated into a second version of the questionnaire that was reviewed by the Delphi panel experts. It was then pilot-tested with a group of 10 emergency physicians. Feedback regarding phraseology and question order was incorporated into the final survey (see Appendix).

We sought a cross-sectional, convenience sample from a potential pool of approximately 300 physicians across the four sites with the goal of obtaining >100 responses. A sample size goal of 100 was instituted for this preliminary survey project convenience sample in order to include approximately 10 subjects per every one survey item. The survey was sent electronically to the four residency listserves from December 2012 to June 2013, with a total of six monthly completion reminder emails.

We collected results electronically and anonymously using SurveyMonkey.com. All data were analyzed using Microsoft Excel. This study was deemed exempt by the local institutional review board.

## RESULTS

**Figure (1a–1d):**
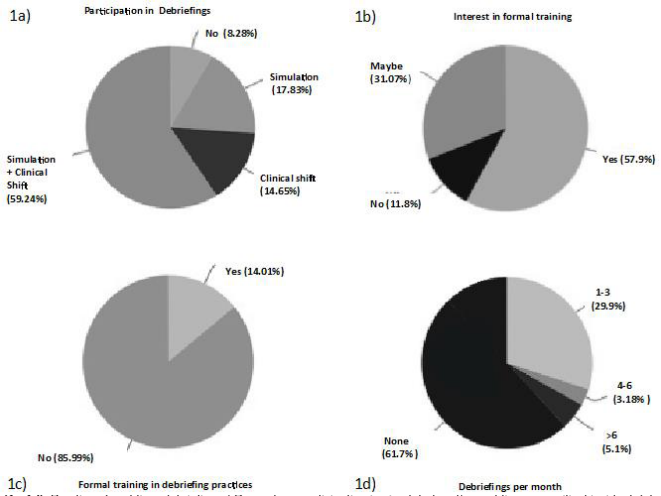
Practice of real-time debriefing a) Percentage participation in simulated and/or real-time non-critical incident debriefings b) Percentage with formal training in debriefing skills c) Percentage expressing interest in formal debriefing training d) Reported percentages of debriefings occurring per month.

We collected 157 responses, representing a response rate between 45% and 52%. Of the respondents, 52% were resident physicians and 47% were attending physicians. No other demographic data were collected. Fifty-nine percent of our respondents reported participating in non-critical incident debriefing* in clinical and simulated settings, whereas 14.6 % reported debriefing only during clinical practice (Figure 1a).

When asked what debriefing meant to physicians, 87.6 % reported that it was a discussion based on real or simulated cases where participants self-reflect and self-analyze their actions and emotions to improve or sustain performance in the future. Other responses are depicted in Table 1a.

With respect to whether respondents had been formally trained in any debriefing technique, only 14% reported affirmatively (Figure 1c). Several comments in this section specified that respondents had learned debriefing skills by watching colleagues or had learned it during simulation debriefing courses. There was significant interest in formal debriefing training in the group surveyed (Figure 1b).

Thirty percent of our respondents reported debriefing on clinical shifts between 1–3 times monthly. Three percent reported debriefing between 4–6 times monthly. The majority of respondents answered less than one debriefing a month (Figure 1d).

Perceived benefits of real-time debriefings are depicted in Table 1e. The majority of respondents indicated that they perceive debriefings to be beneficial for clearing the air after an event (47%), providing feedback to learners and colleagues (66%), identifying knowledge and process gaps (55%), identifying systems errors (55%), promoting of team unity and cohesiveness (37%) and identifying medico-legal ramifications (60%).

With respect to the formats of real-time debriefings conducted, (Figure 1b) 84% of respondents reported that debriefings were performed as a group, while 37.6% reported that debriefings included other professions such as nursing and ancillary staff; 22.9% reported performing individualized debriefings for each learner. Only 15.3% reported inclusion of other specialties, and in the “comments” section several respondent noted that interdisciplinary debriefings were often met with resistance from the other specialties.

Table 1d reflects the different kinds of situations that emergency physicians are most likely to debrief. The majority of respondents reported debriefing about adverse events, near-adverse events, if a colleague was visibly emotionally upset, difficulties during clinical procedures, and miscommunication or poor teamwork; 24.8% reported debriefing after every cardiac code and 25.5 % after every trauma code. One respondent commented that each debriefing was followed up with a personal email to learners to reinforce clinical points learned during debriefings.

Several barriers to real-time medical debriefing were reported by respondents as illustrated in Table 1c; 85.4% reported lack of time during a busy clinical shift as a major deterrent. Other barriers included lack of appropriate training (48.4%), lack of space (35.7%), disinterested colleagues (34.4%) and work environment considerations such as confrontational or defensive co-workers (29.9%). Under “comments” for this question, it was noted by a few respondents that debriefing was not stressed enough in curricula and therefore was often not on the academic physicians’ radar.

## DISCUSSION

Real-time feedback, such as that accomplished through real-time debriefing during a clinical shift in the ED is an important component of a resident physician’s medical education and can have a profound impact on clinical practice.^2^–^5^ Debriefings are significant because they provide a venue for the crucial processes of feedback, reflection and experiential learning that lead to clinical practice pearls for each learner.^7^,^8^

The results from this study confirm that real-time debriefings occur frequently in EDs despite only 14% of respondents reporting formal training in debriefing techniques. The majority of respondents would like formal training, reflecting growing awareness of the potential benefits of real-time debriefing. Although there appears to be a perceived value of the feedback from debriefing, whether there is a proven benefit to patient care, morbidity, mortality and learner education is difficult to pinpoint and remains to be investigated. Any potential pitfalls of real-time debriefing, such as medico-legal ramifications or unstable work environment as a consequence of debriefing, also remain to be elucidated. It would also be interesting and likely beneficial to study the effects of instituting a department-wide debriefing protocol on learner education, staff interaction and systems/process improvement. The effect of non-critical incident debriefing on patient safety is another potential area of research. Finally, as there is little clarity on the format of debriefing techniques being used it would be enlightening to investigate which kind of debriefing occurs in the ED environment.

Simulation debriefing is based on Kolb’s principles of experiential learning.^15^ Kolb’s cycle of experiential learning is based on learners’ experiencing a particular event, reflecting on that event, conceptualizing it abstractly and actively experimenting with their newly conceptualized knowledge. Experiential learning occurs in clinical practice during medical student clerkships, residency and beyond. Learners experience a particular clinical case and they reflect on the management of the case. Learners then conceptualize the knowledge and use it when seeing a similar case in the future.^15^ The assumption in this picture is that learners perform this learning cycle independently. While it may be true for some learners, a facilitated approach to reflection and conceptualization may aide in the learning process. Non-critical incident debriefing can be viewed as the facilitation of experiential learning in real time. It can be tailored to complex clinical cases or events. It can be applied to a diverse set of learners, focusing on learner-specific knowledge, process or procedural gaps. When involving other disciplines and professions it can also pave the way for effective teamwork. In these ways, real-time, non-critical incident debriefing has the potential to address some of the barriers to effective bedside teaching in the academic and non-academic ED mentioned before.^1^

## LIMITATIONS

This study is limited by the nature of any survey-based project and the potential biases introduced by self-reporting. Further, it is limited by the limited response rate. In addition, the survey data provide only a brief glimpse into the practice patterns and trends relating to debriefings in academic EDs in one metropolitan city, which may lead to regional bias and may not allow for generalization to national characteristics of this phenomenon.

## CONCLUSION

This survey regarding the practice of real-time, non-critical incident debriefings in four major academic emergency programs within New York City sheds light on three major, pertinent points: 1) Real-time, non-critical incident debriefing definitely occurs in clinical emergency practice; 2) in general, real-time debriefing is perceived to be of some value with respect to education, systems and performance improvement; 3) although being practiced by clinicians, most report no formal training in actual debriefing techniques. In conclusion, further studies are needed to clarify actual benefits of real-time, non-critical incident debriefing as well as details on potential pitfalls of this practice and recommendations for best practices for use.

## Supplementary Information



## Figures and Tables

**Table:** Characteristics of real-time debriefing as perceived and understood by emergency physicians.

Characteristics of Real-Time Debriefing Practices	Percentage responses (n)
1a. Emergency physicians’ understanding of “debriefing”	
i) A discussion based on a real or simulated case scenario about its management.	45.9 (72)
ii) A post-medical error discussion at an administrative level such as Root Cause Analysis/or morbidity and mortality Conference	12.7 (20)
iii) A discussion, based on real or simulated cases, aimed at identifying knowledge or performance gaps	51.6 (81)
iv) A discussion, based on real or simulated cases, where participants self-reflect and analyze their actions and emotions, to improve or sustain performance in the future	87.9 (138)
1b. Formats of real-time debriefings being performed	
i) Separately for each individual learner	22.9 (36)
ii) Group of learners (residents or medical students)	84.1 (132)
iii) Inter-professional (with nursing and/or ancillary support staff)	37.6 (59)
iv) Interdisciplinary	15.3 (24)
v) Initially as a group followed by individually for learners	13.4 (21)
1c. Perceived barriers to real-time debriefing	
i) A lack of training in debriefing skills	48.4 (76)
ii) Time constraints	85.4 (134)
iii) Disinterested colleagues	34.4 (56)
iv) Lack of appropriate space	35.7 (54)
v) Work environment considerations (emotional/defensive/confrontational co-workers)	29.9 (47)
1d. Situations most likely to be debriefed	
i) Emotionally upset colleagues	66.2 (104)
ii) Adverse event	68.8 (108)
iii) Near-adverse event	59.2 (93)
iv) Difficulties in clinical procedure performance	59.2 (93)
v) Miscommunications and poor teamwork	65.6 (103)
vi) Emotionally charged resuscitations	58.0 (91)
vii) All cardiac codes	24.8 (39)
viii) All trauma codes	25.5 (40)
ix) All of the above	24.8 (39)
1e. Perceived benefits of real-time debriefings	
i) Clears the air	42.0 (66)
ii) Provides a venue for learner and colleague feedback.	65.6 (103)
iii) Provides a venue for addressing learner and colleague knowledge and/or performance gaps	54.8 (86)
iv) Promotes team cohesiveness and unity with respect to patient care	55.4 (87)
v) Provides opportunity for discussion of the medico-legal ramifications of adverse or near-adverse events	15.9 (25)
vi) Identifies systems errors leading to systems-process improvements	59.8 (94)
vii) All of the above	36.9 (58)

## Footnotes

* “Critical incident debriefing” or “critical incident stress debriefing” are well established terms in psychological literature, that refer to a deliberate counseling method designed to mitigate the stress response generated from emotionally traumatic cases or “critical incidents” such as pediatric deaths or mass casualty events.^17^ As “critical incident debriefing” focuses on stress mitigation and not education, process or systems improvement, it was excluded from the literature search.
