# Supplementary material for: Characteristics of Real-Time, Non-Critical Incident Debriefing Practices in the Emergency Department
Source: West J Emerg Med. 2016 Dec 5;18(1):146–51. doi: 10.5811/westjem.2016.10.31467 (PMC5226751; doi:10.5811/westjem.2016.10.31467)
Supplement: Supplementary file 1 [file wjem-18-146-s001.docx]

**APPENDIX**

**Questionnaire on Real-time Debriefing Practices.**

1. What is your medical profession? a) Physician b) Resident Physician c) Registered Nurse d) Other (please specify)

2. Have you participated in or conducted any debriefing sessions during simulations or clinical shifts?

a) No b) Yes, post simulation sessions only c) Yes, in real-time during clinical practice d) Yes both post simulation and during clinical practice

3. Have you any formal training (such as dedicated course or workshop) in the practice of debriefing techniques/models?

a) Yes b) No

If yes please indicate in the comment box which kinds of debriefing techniques/models.

4. If no to the above question, would you like to obtain formal training in debriefing techniques?

a) Yes b) No c) Maybe

5. What is your understanding of “Debriefings?” Please select all that apply:

a) A discussion, based on real or simulated case scenario, specifically about case

scenario management

b) A post-medical error discussion at administrative level such as Root Cause Analysis/ Morbidity Mortality Conference

c) A discussion, based on real or simulated cases, aimed at identifying knowledge or performance gaps

d) A discussion based on real or simulated cases, where participants self-reflect and analyze their actions and emotions to improve or sustain performance in the future.

e) None of the above

f) Other (Please explain in the comment section below)

6. How many real-time debriefings do you participate in or conduct a month? (Real-time debriefings refer to debriefings that occur during or immediately after a clinical shift)

a) 1-3 b) 4-6 c) More than 6 d) None

7. Please select all that have applied: In your experience of real-time debriefings, the format for these debriefings were:

a) Performed separately for each individual learner

b) Performed as a group of learners (residents or medical students)

c) Incorporated other professions such as nursing or ancillary support staff

d) Included other disciplines such as surgery, internal medicine

e) Performed first as a group and then individually for learners

f) None of the above

g) Other (please provide examples of your personal real-time debriefings)

8. What do you consider to be some barriers to real-time debriefing? Please select all that may apply:

a) There is a lack of training for effective debriefing in real-time

b) There is not enough time

c) There is often a lack of interest by colleagues

d) There is no appropriate space

e) It can lead to situations where people can become emotional/defensive/confrontations thus potentially corrupting the work environment

f) Other – please provide suggestions/

9. Which of the following situations would you be most likely to debrief in real-time? Please select all that may apply:

a) Visibly emotionally shaken healthcare worker

b) Noted adverse events during patient management

c) Noted near adverse events (not an actual error but a near error)

d) Noted difficulties in clinical procedure performance

e) Noted miscommunications and poor teamwork during patient management

f) An emotionally charged resuscitation of certain patient groups such as pediatric, young adult or a patient known to you.

g) Every cardiac code

h) Every trauma code

i) All of the above

j) Other – please provide examples

10. In your opinion what does real-time debriefing afford? Please select all that may apply:

a) It is a way to clear the air after a code type situation

b) It is a venue to provide learner and colleague feedback

c) It is a means of addressing learner and colleague knowledge and/or performance gaps

d) Discussion of incidents promotes team cohesiveness and unity with respect to patient care

e) Debriefing of adverse events or near adverse events can have medico-legal ramifications

f) Debriefing can lead to identification of systems errors, which can contribute to system/process improvements

g) All of the above

h) Other. Please explain below.
